# Pseudoachondroplasia and Multiple Epiphyseal Dysplasia: A 7-Year Comprehensive Analysis of the Known Disease Genes Identify Novel and Recurrent Mutations and Provides an Accurate Assessment of Their Relative Contribution

**DOI:** 10.1002/humu.21611

**Published:** 2011-09-15

**Authors:** Gail C Jackson, Laureane Mittaz-Crettol, Jacqueline A Taylor, Geert R Mortier, Juergen Spranger, Bernhard Zabel, Martine Le Merrer, Valerie Cormier-Daire, Christine M Hall, Amaka Offiah, Michael J Wright, Ravi Savarirayan, Gen Nishimura, Simon C Ramsden, Rob Elles, Luisa Bonafe, Andrea Superti-Furga, Sheila Unger, Andreas Zankl, Michael D Briggs

**Affiliations:** 1 Wellcome Trust Centre for Cell Matrix Research, University of ManchesterManchester, United Kingdom; 2 National Genetics Reference LaboratoryManchester, United Kingdom; 3 Centre Hospitalier Universitaire VaudoisLausanne, Switzerland; 4 Department of Medical Genetics, Antwerp University HospitalAntwerp, Belgium; 5 Institute for Human Genetics and Center for Paediatrics and Adolescent MedicineFreiburg, Germany; 6 Hôpital Necker-Enfants MaladesParis, France; 7 Great Ormond Street Hospital for ChildrenLondon, United Kingdom; 8 Sheffield Children's HospitalSheffield, United Kingdom; 9 Institute of Human GeneticsNewcastle-upon-Tyne, United Kingdom; 10 Murdoch Children's Research Institute, Genetic Health Services Victoria and Department of Paediatrics, University of MelbourneMelbourne, Australia; 11 Department of Paediatric Imaging, Tokyo Metropolitan Children's Medical CentreJapan; 12 Bone Dysplasia Research Group, University of Queensland Centre for Clinical Research, University of QueenslandBrisbane, Australia

**Keywords:** pseudoachondroplasia, multiple epiphyseal dysplasia, COMP, SLC26A2

## Abstract

Pseudoachondroplasia (PSACH) and multiple epiphyseal dysplasia (MED) are relatively common skeletal dysplasias resulting in short-limbed dwarfism, joint pain, and stiffness. PSACH and the largest proportion of autosomal dominant MED (AD-MED) results from mutations in cartilage oligomeric matrix protein (*COMP*); however, AD-MED is genetically heterogenous and can also result from mutations in matrilin-3 (*MATN3*) and type IX collagen (*COL9A1*, *COL9A2*, and *COL9A3*). In contrast, autosomal recessive MED (rMED) appears to result exclusively from mutations in sulphate transporter solute carrier family 26 (*SLC26A2*). The diagnosis of PSACH and MED can be difficult for the nonexpert due to various complications and similarities with other related diseases and often mutation analysis is requested to either confirm or exclude the diagnosis. Since 2003, the European Skeletal Dysplasia Network (ESDN) has used an on-line review system to efficiently diagnose cases referred to the network prior to mutation analysis. In this study, we present the molecular findings in 130 patients referred to ESDN, which includes the identification of novel and recurrent mutations in over 100 patients. Furthermore, this study provides the first indication of the relative contribution of each gene and confirms that they account for the majority of PSACH and MED. Hum Mutat 33:144–157, 2012. © 2011 Wiley Periodicals, Inc.

## Introduction

Pseudoachondroplasia (PSACH; MIM# 177170) and multiple epiphyseal dysplasia (MED; MIM# 132400) are relatively common skeletal dysplasias that can be inherited as either autosomal dominant (PSACH and AD-MED) or recessive (AR-MED; rMED) conditions [Briggs and Chapman, 2002; Superti-Furga and Unger, 2007].

PSACH usually manifests in the second year of life and is characterized by moderate to severe disproportionate short stature, ligamentous laxity, and degenerative joint disease. MED is a clinically variable disease that manifests in early-to-mid childhood with joint pain and stiffness, mild to moderate short stature, and early onset osteoarthritis [Barrie et al., 1958; Fairbank, 1947; Rimoin et al., 1994]. At least one other disorder overlaps phenotypically with MED; familial hip dysplasia (Beukes type; MIM# 142669) [Cilliers and Beighton, 1990], which is mapped to chromosome 4q35 [Roby et al., 1999] and has been grouped with AD-MED in the 2010 revision of the “International Nosology and Classification of Genetic Skeletal Disorders” [Warman et al., 2011].

PSACH is believed to result exclusively from mutations in the gene encoding cartilage oligomeric matrix protein (*COMP*; MIM# 600310), as does the largest proportion of AD-MED [Briggs and Chapman, 2002; Briggs et al., 1995; Hecht et al., 1995]. Presumed autosomal recessive forms of PSACH [Dennis and Renton, 1975; Wynne-Davies et al., 1986; Young and Moore, 1985] were proposed to be caused by germline mosaicism and this has been proven by *COMP* analysis [Ferguson et al., 1997; Hall et al., 1987]. However, a disorder resembling PSACH without a *COMP* mutation has been described [Spranger et al., 2005], but the genetic basis of this PSACH variant remains undetermined.

AD-MED is a much more heterogeneous disorder, both at the phenotypic and genetic levels. In addition to *COMP* mutations, it can also result from mutations in the genes encoding matrilin-3 (*MATN3*, EDM5; MIM# 607078) and type IX collagen (*COL9A1*, EDM6; MIM# 120210; *COL9A2*, EDM2; MIM# 600204; *COL9A3*, EDM3; MIM# 600969, respectively) [Briggs and Chapman, 2002; Chapman et al., 2001; Czarny-Ratajczak et al., 2001; Muragaki et al., 1996; Paassilta et al., 1999; Unger et al., 2008]. Furthermore, several studies have suggested that a variable proportion of AD-MED can result from mutations in other genes [Jakkula et al., 2005; Zankl et al., 2007], but the identities of these genes have not yet been determined. AR-MED (rMED) can result from homozygosity or compound heterozygosity for mutations in the gene encoding *SLC26A2* (EDM4; MIM# 226900) [Hastbacka et al., 1999; Rossi and Superti-Furga, 2001; Superti-Furga et al., 1999] and is the mild end of the phenotypic spectrum that includes achondrogenesis 1B and diastrophic dysplasia [Rossi and Superi-Furga, 2001].

The extensive genetic heterogeneity of MED combined with wide-ranging clinical variability, including both intra- and interfamilial variability, and various complications such as osteochondritis dissecans and mild myopathy provide a diagnostic challenge for the nonexpert [Makitie et al., 2004; Newman et al., 2000; Unger, 2002; Unger et al., 2008; Zankl et al., 2007]. In order to better understand the molecular genetics of MED, we screened for *COMP*, *COL9A1, COL9A2, COL9A3, MATN3,* and *SLC26A2* mutations in over 100 patients referred to the European Skeletal Dysplasia Network (ESDN) via the on-line case manager (www.ESDN.org). In many of these patients, a clinical diagnosis of PSACH or MED was confirmed (or suspected) by the expert panel of the ESDN prior to mutation screening. However, we also included a cohort of patients, which the expert panel felt were not classical examples of these diseases due to a variety of unusual clinical and/or radiographic features (detailed in Supp. Table S1). Indeed, in many of these cases an alternative diagnosis was suggested prior to mutation screening. However, the inclusion of these patients was important for identifying phenotypic outliers of the “classical” PSACH and MED disease spectrum and to also identify specific radiographic and/or clinical features that are generally uncharacteristic of molecularly confirmed MED.

## Materials and Methods

All cases were submitted on-line via the secure case manager site (https://cm.esdn.org/). Every case was then reviewed and discussed by the ESDN panel members. Following discussion, DNA samples from the patient, and when available affected and unaffected family members, were sent for mutation screening in Manchester (for PSACH and AD-MED) or Lausanne (AR-MED). Screening of *COMP* (exons 8–19), *MATN3* (exon 3), and the type IX collagen genes (only the exon sequence and splice donor/acceptor sites of exon 8 of *COL9A1* and exon 3 of *COL9A2* and *COL9A3*) was performed as previously described [Jackson et al., 2004; Kennedy et al., 2005a; Zankl et al., 2007]. This screening protocol reflected our current knowledge of all known locations of PSACH and AD-MED mutations in the type III repeat and C-terminal regions of COMP, the A-domain of *MATN3*, and the COL3 domain of type IX collagen. Screening of *SLC26A2* was performed as previously described [Rossi and Superti-Furga, 2001]. All mutations were confirmed in a second PCR reaction. Primer sequences and PCR conditions for exons 1–7 of *COMP*, exons 3–6 of *MATN3*, exons 1–3 and 5–6 of *MATN1*, exons 2, 6+7 of *MATN4*, and exon 50 of *COL2A1* are presented in Supp. Table S2. These exons encode important structural and/or functional domains in COMP (type II EGF-like repeats), *MATN3* (EGF-like repeats), matrilin-1 (A-domains), matrilin-4 (A-domains), and type II collagen (triple-helical region).

Proof of pathogenicity was defined by one or more of the following criteria; (1) a previously published mutation with co-segregation in a family and/or absent in controls, (2) a *de novo* mutation or co-segregation in this study, (3) alteration of an evolutionary conserved known functional residue in either the N-type motif or C-type motif of the type III repeat region of COMP or the A-domain of *MATN3*, (4) biochemical evidence of a pathogenetic affect.

## Results

As part of this 7-year study (2003–2010), we screened DNA from 28 PSACH patients for mutations in *COMP*, 77 patients (suspected AD-MED and variants) for mutations in *COMP*, *MATN3*, and the three type IX collagen genes (*COL9A1*, *COL9A2*, and *COL9A3*), and 22 patients for mutations in *SLC26A2* (suspected rMED).

### Mutation Analysis of COMP in Suspected PSACH

COMP is a modular protein comprising an amino-terminal coiled-coil oligomerization domain, four type II (EGF-like) domains, seven type III (CaM-like) repeats, and a C-terminal globular domain (CTD).

**Figure 1:**
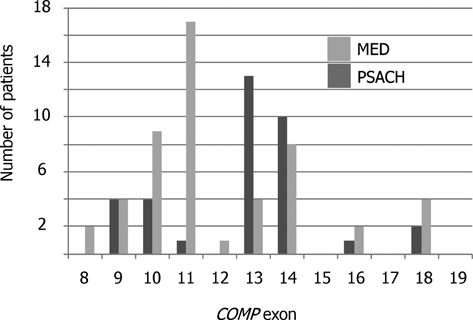
Exon distribution of COMP missense mutations in PSACH and MED. The cumulative distribution of COMP missense mutations from this study and that published by Kennedy et al. [2005a] is represented graphically. The total number of patients reported in these two studies is 86 (*n* = 35 PSACH; *n* = 51 MED) and these data clearly show that exons 10 and 11 are enriched for MED missense mutations, while missense mutations in exon 13 mostly cause PSACH. In these two studies, we identified no COMP missense mutations in exons 15 (aa 557–572), 17 (aa 639–696), and 19 (aa 743–757) and only a single MED missense mutation in exon 12.

**Figure 2:**
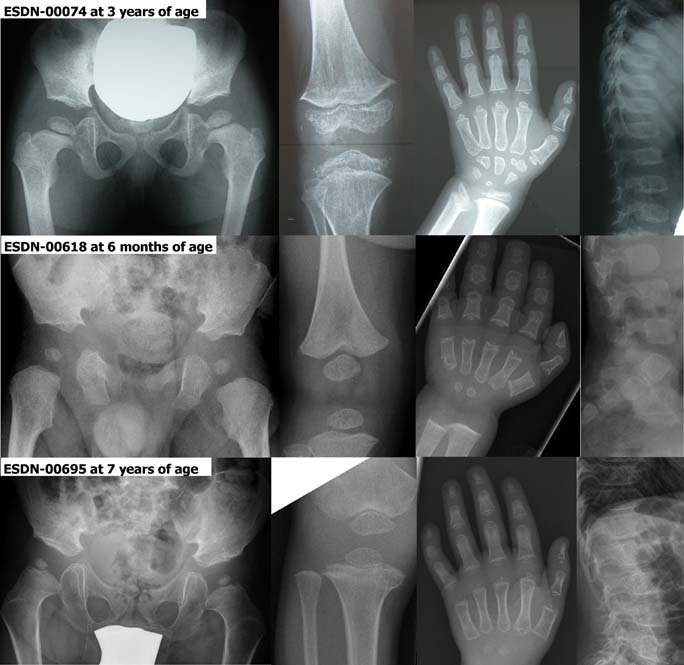
Radiographic findings in *COMP* negative patients referred as PSACH. ESDN-00074: Radiographs of the spine, pelvis, knees, and left hand. With the exception of the left hand taken at 4 years of age, all radiographs were taken at the age of 3 years. The vertebral bodies are flattened, the proximal femoral epiphyses are small, and the femoral necks are short. The trochanters minor are well ossified and prominent present. The knee epiphyses are small and irregularly ossified. The metaphyses in the knees are widened and the femoral distal metaphyses have spikes at both ends. The submetaphyseal regions have a striated pattern. The hands show shortening and broadening of the metacarpals and phalanges with small epiphyses. The epiphyses of the proximal phalanges are fragmented. There is advanced carpal ossification with rather rectangular (and not rounded) shape of the carpal bones. The distal ulna shows precocious ossification of the epiphysis and cupped metaphysis. ESDN-00618: Radiographs of spine, pelvis, knee, and hand taken at the age of 6 months. The pelvis is abnormal with flat and trident acetabular roof and small and broad iliac wings. The ischiadic bones are broad. The proximal femoral epiphyses are well ossified for age. The femoral necks appear broad. The hand shows shortening of phalanges and metacarpals, especially the proximal and middle phalanges are very short with poor diaphyseal modeling and precocious ossification of the epiphyses that are attached to the metaphysis. The vertebral bodies are mildly foreshortened with posterior scalloping. No gross abnormalities are seen at the knee. ESDN-00695: Radiographs of spine, pelvis, knee, and hand taken at the age of 7 years. The mini-epiphyses in the hips and the small epiphyses in the knees with translucent submetaphyseal areas in the proximal tibia are reminiscent of PSACH. However, the hand shows only mild shortening of the phalanges and metacarpals. In addition, there is marked delay in carpal ossification. The epiphyses in the wrist and hands are too small for age. The vertebral bodies are flattened and elongated.

We identified type III repeat region *COMP* mutations in 27 of the 28 patients with PSACH (>96%; Table 1; Fig. 1), which were distributed between seven exons (exons 9, 10, 11, 13, 14, 16, and 18) and comprised missense mutations (67%) or small deletions (30%) and deletions/insertions (3%). We did not identify any PSACH missense mutations in exons 8, 12, 15, 17, or 19 of *COMP*, which is consistent with our previous findings [Kennedy et al., 2005a] (Fig. 1), although the biological significance of this observation remains unknown. Ten of the mutations (37%) were novel while the common p.Asp473del mutation was identified in six patients (22%). The CTD mutations p.Thr529Ile, pGly719Ser, and p.Thr585Arg, which we and others have previously described [Briggs et al., 1998; Jakkula et al., 2003; Kennedy et al., 2005b], were identified in four patients thus confirming the clustering of the CTD mutations into distinct regions [Kennedy et al., 2005b]. We also screened *COMP* in three patients with atypical PSACH but did not identify a mutation (Table 2; Fig. 2).

**Table 1:** *COMP* Mutations Identified in 27 Patients with Clinical and Radiographically Confirmed PSACH

Patient/family	Diagnosis on referral^a^	Diagnosis following review^b^	Exon	DNA change	Protein change	COMP domain	Published and/or proof of pathogenicity
ESDN-00814	PSACH	PSACH	9	c.869A>G	p.Asp290Gly	T3	Related mutation p.Asp290Asn shown to be de novo in Ikegawa et al. [1998]
ESDN-00877	PSACH	PSACH	9	c.895G>C	p.Gly299Arg	T3	Ikegawa et al. [1998] (absent in controls)
ESDN-00385	PSACH	PSACH	10	c.976G>T	p.Asp326Tyr	T3	de novo mutation in this family and conserved functional residue in C-type motif
ESDN-00622	PSACH	PSACH	10	c.1021_1026del	p.Glu341_Asp342del	T3	Kennedy et al. [2005a] and de novo mutation in this family
ESDN-00155	PSACH	n/d	10	c.1048_1116del	p.Asn350_Asp372del	T3	de novo mutation in this family and deletion of conserved functional residues in C- and N-type motifs
ESDN-00966	PSACH	PSACH	10	c.1133A>T	p.Asp378Val	T3	de novo mutation in this family
ESDN-00672	MED Fairbank	PSACH	11	c.1159T>C	p.Cys387Arg	T3	Conserved functional residue in C-type motif
ESDN-01201	PSACH	PSACH	11	c.1205_1212delinsTCTGT	p.Gly402_Gly404delinsValCys	T3	Deletion of conserved functional residues in C-type motif
ESDN-00197	PSACH	PSACH	13	c.1318G>A	p.Gly440Arg	T3	Loughlin et al. [1998] (absent in controls)
ESDN-00294	SMD	PSACH	13	c.1336G>A	p.Asp446Asn	T3	Maddox et al. [1997] (absent in controls) and de novo mutation in this family
ESDN-01016	No diagnosis	PSACH	13	c.1343G>C	p.Cys448Ser	T3	Conserved functional residue in C-type motif
ESDN-00165	PSACH	PSACH	13	c.1417_1419del	p.Asp473del	T3	Hecht et al. [1995] (common mutation)
ESDN-00166	PSACH	PSACH	13	c.1417_1419del	p.Asp473del	T3	Hecht et al. [1995] (common mutation)
ESDN-00449	PSACH	PSACH	13	c.1417_1419del	p.Asp473del	T3	Hecht et al. [1995] (common mutation)
ESDN-00658	PSACH	PSACH	13	c.1417_1419del	p.Asp473del	T3	Hecht et al. [1995] (common mutation)
ESDN-00724	PSACH	PSACH	13	c.1417_1419del	p.Asp473del	T3	Hecht et al. [1995] (common mutation)
ESDN-01015	PSACH	PSACH	13	c.1417_1419del	p.Asp473del	T3	Hecht et al. [1995] (common mutation)
ESDN-00242	PSACH	PSACH	13	c.1417G>C	p.Asp473His	T3	Conserved functional residue in C-type motif
ESDN-00020	PSACH	PSACH	13	c.1423G>A	p.Asp475Asn	T3	Deere et al. [1998] (absent in controls) and conserved functional residue in C-type motif
ESDN-00248	PSACH	PSACH	13	c.1445A>G	p.Asp482Gly	T3	Susic et al. [1998] and conserved functional residue in C-type motif
ESDN-00204	PSACH	PSACH	14	c.1520A>G	p.Asp507Gly	T3	Deere et al. [1998] (absent in controls) and conserved functional residue
ESDN-00490	PSACH	PSACH	14	c.1532A>G	p.Asp511Gly	T3	Conserved functional residue in C-type motif
ESDN-01203	No diagnosis	PSACH	14	c.1544A>G	p.Asp515Gly	T3	de novo mutation in this family
ESDN-00034	PSACH	PSACH	14	c.1586C>T	p.Thr529Ile	CTD	Kennedy et al. [2005a,b] (absent in controls)
ESDN-00575	PSACH	PSACH	14	c.1586C>T	p.Thr529Ile	CTD	Kennedy et al. [2005a,b] (absent in controls)
ESDN-00109	PSACH	Mild PSACH or MED	16	c.1754C>G	p.Thr585Arg	CTD	Briggs et al. [1998] (family studies)
ESDN-00894	PSACH	PSACH	18	c.2155G>A	p.Gly719Ser	CTD	Kennedy et al. [2005a,b] (absent in controls)

a Diagnosis as provided by the referring clinician.

b Consensus reached by the ESDN panel after review.

Proof of pathogenicity is defined by one or more of the following criteria; (1) a previously published mutation with family studies or absent in controls (indicated by parenthesis), (2) a de novo mutation or co-segregation in this study, (3) alteration of an evolutionary conserved functional residue in either the N-type motif or C-type motif of the type III repeat region of COMP. Nucleotide numbering according to cDNA sequence with GenBank accession number NM_000095.2. Nucleotide 1 has been counted as the first nucleotide of the translation initiation codon.

PSACH, pseudoachondroplasia; MED, multiple epiphyseal dysplasia; SMD, spondylometaphyseal dysplasia; T3, type 3 repeat region of COMP; CTD, C-terminal domain of COMP; n/d, diagnosis not discussed by ESDN.

**2:** Three Patients Screened for *COMP* Mutations that had (S)EMD or Nontypical PSACH

Patient	Diagnosis on referral^a^	Reasons why not “classical” PSACH	Alternative diagnosis suggested prior to mutation screening^b^
ESDN-00074	(S)EMD unspecified	(1) Advanced carpal ossification.	(1) SEMD unspecified
		(2) Flat instead of rounded vertebrae.	
		(3) No mini-epiphyses in the hips.	
ESDN-00618	PSACH	(1) Radiographic features were not severe enough in knees, hips, and spine.	(1) Acromesomelic dysplasia
		(2) Hand radiographs show very short and broad phalanges with precocious ossification of the epiphyses attached to the metaphyses.	(2) Acrocapitofemoral dysplasia(3) CHH
ESDN-00695	PSACH	(1) Vertebral bodies appeared too flat for PSACH but instead resembled those in the non-Comp pPSACH family (14).	(1) AR-PSACH(2) pPSACH
		(2) Hips and knees are reminiscent of AD PSACH.	(3) SED
		(3) Tubular bones in hands are not short enough and the delayed carpal ossification is too pronounced.	

a Diagnosis as provided by the referring clinician.

b Diagnosis suggested by the ESDN panel. ESDN-00695 had previously tested negative for a *COL2A1* mutation.

PSACH, pseudoachondroplasia; SED, spondyloepiphyseal dysplasia; (S)EMD, (spondylo)-epi-metaphyseal dysplasia; CHH, cartilage hair hypoplasia; AR-PSACH, autosomal recessive PSACH; pPSACH, pseudo-PSACH.

### Mutation Analysis of COMP, MATN3, and the Type IX Collagen Genes in Suspected AD MED

We identified *COMP* mutations in 37 patients with MED (Table 3), which were distributed between nine exons (exons 8–14, 16, and 18) and comprised missense mutations (>86%), small in-frame deletions (∼5%), duplications (∼5%), insertions (<3%), and deletion/insertions (<3%). We did not identify any MED missense mutations in *COMP* exons 15, 17, and 19, which are again consistent with our previous findings [Kennedy et al., 2005a] (Fig. 1). Fifteen (∼40%) of the mutations were novel, while the recurrent mutations p.Asp385Asn, p.Asn523Lys, and p.Arg718Pro/Trp were each identified in three patients [Ballo et al., 1997; Kennedy et al., 2005b; Mabuchi et al., 2003]. Interestingly, MED patient ESDN-00594 was found to have two potential *COMP* missense mutations; p.Gly501Asp in the Type III-repeat region and p.Gln756Arg in the CTD (Table 3).

**Table 3:** *COMP*, *MATN3*, *COL9A2*, *COL9A3,* and *SLC26A2* Mutations Identified in 53 Patients with Clinical and Radiographically Confirmed MED

Patient/family	Diagnosis on referral^a^	Diagnosis following review^b^	Gene	Exon	DNA mutation	Protein change	Domain	Published and/or proof of pathogenicity
ESDN-00596	MED	MED	*COMP*	8	c.827C>G	p.Pro276Arg	T3	Czarny-Ratajczak et al. [2001] (family studies and absent in controls)
ESDN-00016^Z^	MED	Mild PSACH or MED	*COMP*	9	c.893C>T	p.Ser298Leu	T3	Kennedy et al. [2005a] and de novo mutation in this family
ESDN-00815	MED	MED	*COMP*	9	c.932C>A	p.Ala311Asp	T3	de novo mutation in this family and conserved functional residue in N-type motif
ESDN-00809	MED	Nontypical MED^c^	*COMP*	9	c.950A>G	p.Asp317Gly	T3	Conserved functional residue in C-type motif
ESDN-00461	Polyepiphyseal dysplasia	MED	*COMP*	10	c.977A>G	p.Asp326Gly	T3	de novo mutation in this family and conserved functional residue in C-type motif
ESDN-01020	MED	MED	*COMP*	10	c.1043G>T	p.Cys348Phe	T3	Family studies and conserved functional residue in C-type motif
ESDN-00053^Z^	MED	MED	*COMP*	10	c.1111T>A	p.Cys371Ser	T3	Conserved functional residue in N-type motif
ESDN-01112	PSACH	MED	*COMP*	10	c.1112G>A	p.Cys371Tyr	T3	Susic et al. [1997] (absent in controls) and conserved functional residue in N-type motif
ESDN-00172^Z^	MED	MED	*COMP*	10	c.1120G>A	p.Asp374Asn	T3	Zankl et al. [2007], de novo mutation in this family and conserved functional residue in C-type motif
ESDN-00107^Z^	SED or MED	MED	*COMP*	10	c.1126G>A	p.Asp376Asn	T3	Zankl et al. [2007] and conserved functional residue in C-type motif
ESDN-00049^Z^	MED	Nontypical MED^c^	*COMP*	11	c.1153G>A	p.Asp385Asn	T3	Mabuchi et al. [2003] and conserved functional residue in C-type motif
ESDN-00509	MED	MED	*COMP*	11	c.1153G>A	p.Asp385Asn	T3	Mabuchi et al. [2003] and conserved functional residue in C-type motif
ESDN-00597	MED	MED	*COMP*	11	c.1153G>A	p.Asp385Asn	T3	Mabuchi et al. [2003], family studies and conserved functional residue in C-type motif
ESDN-00032	MED	MED	*COMP*	11	c.1152_1154delCGA	p.Asp385del	T3	Kennedy et al. [2005a] and deletion of conserved functional residue in C-type motif
ESDN-00323	MED	Nontypical MED^c^	*COMP*	11	c.1153G>T	p.Asp385Tyr	T3	Family studies and conserved functional residue in C-type motif
ESDN-01120	MED	MED	*COMP*	11	c.1189G>C	p.Asp397His	T3	de novo mutation in this family and conserved functional residue in C-type motif
ESDN-01116	MED	MED	*COMP*	11	c.1210G>A	p.Gly404Arg	T3	de novo mutation in this family and conserved functional residue in C-type motif
ESDN-00094^Z^	PSACH-MED	MED	*COMP*	11	c.1229G>A	p.Cys410Tyr	T3	Zankl et al. [2007] and conserved functional residue in C-type motif
ESDN-00595^Z^	PSACH-MED	Mild PSACH or MED	*COMP*	11	c.1229G>A	p.Cys410Tyr	T3	Zankl et al. [2007] and conserved functional residue in C-type motif
ESDN-00089^Z^	MED	MED	*COMP*	11	c.1245C>G	p.Asn415Lys	T3	Zankl et al. [2007], de novo mutation in this family and conserved functional residue in C-type motif
ESDN-00227	MED	MED	*COMP*	11	c.1245C>G	p.Asn415Lys	T3	Zankl et al. [2007] and conserved functional residue in C-type motif
ESDN-00125^Z^	MED	MED	*COMP*	12	c.1280G>A	p.Gly427Glu	T3	Deere et al. [1998] (absent in controls) and conserved functional residue in N -type motif
ESDN-00191	MED	MED	*COMP*	12	c.1289_1294del(GTGACA)ins(TGTGGT)	p.Cys430_Ser432del insLeuTrpCys	T3	Deletion of conserved functional residues in N-type motif
ESDN-00430	MED with neuropathy	MED	*COMP*	13	c.1371_1373del	p.Glu457del	T3	Newman et al. [2000] and Mabuchi et al. [2003] (de novo) and deletion of conserved functional residue
ESDN-00068^Z^	MED	MED	*COMP*	13	c.1419_1420insGAC	p.Asp473_Asn474 insAsp	T3	Zankl et al. [2007] and del/ins of conserved functional residues in C-type motif
ESDN-00871	MED	MED	*COMP*	13	c.1417_1419dupGAC	p.Asp473dup	T3	Delot et al. [1999] (family studies) and conserved functional residues in C-type motif
ESDN-00907	MED	MED	*COMP*	13	c.1417_1419dupGAC	p.Asp473dup	T3	Delot et al. [1999] (family studies) and conserved functional residues in C-type motif
ESDN-00422	MED or SEMD-JL	MED	*COMP*	14	c.1502G>A	p.Gly501Asp	T3	Conserved functional residue in N-type motif
ESDN-00359	rMED	MED	*COMP*	14	c.1502G>Ac.1504G>T	p.Gly501Aspp.Asp502Tyr	T3	Conserved functional residues in N-type motif
ESDN-00594^d^	MED	MED	*COMP*	14	c.1502G>A	p.Gly501Asp	T3	Conserved functional residue in N-type motif
ESDN-00123^Z^	MED	MED	*COMP*	14	c.1569C>G	p.Asn523Lys	T3	Ballo et al. [1997] (family study)
ESDN-00382	MED sacroiliitis	MED	*COMP*	14	c.1569C>G	p.Asn523Lys	T3	Ballo et al. [1997] (family study)
ESDN-00751	MED	MED or type II collagen	*COMP*	14	c.1569C>G	p.Asn523Lys	T3	Ballo et al. [1997] (family study)
ESDN-00752	MED	MED	*COMP*	16	c.1754C>T	p.Thr585Met	CTD	Briggs et al. [1998] (family study)
ESDN-00336	Mild PSACH/MED	MED	*COMP*	18	c.2153G>C	p.Arg718Pro	CTD	Kennedy et al. [2005a,b] (absent in controls) and de novo mutation in this family
ESDN-00080^Z^	MED	MED	*COMP*	18	c.2152C>T	p.Arg718Trp	CTD	Mabuchi et al. [2003] (absent in controls) and Kennedy et al. [2005a,b] (absent in controls)
ESDN-00066	MED	MED	*COMP*	18	c.2152C>T	p.Arg718Trp	CTD	Mabuchi et al. [2003] (absent in controls) and Kennedy et al. [2005a,b] (absent in controls)
ESDN-00594^d^	MED	MED	*COMP*	18	c.2267A>G	p.Gln756Argno change	CTD	No
					c.2274+1g>c		3′ UTR	No

ESDN-00590	MED	MED	*MATN3*	2	c.359C>T	p.Thr120Met	βB	Jackson et al. [2004] (family studies and absent in controls) and co-segregation in this family
ESDN-00903	MED	MED	*MATN3*	2	c.359C>T	p.Thr120Met	βB	Jackson et al. [2004] (family studies and absent in controls) and co-segregation in this family
ESDN-00003	MED	rMED	*MATN3*	2	c.361C>T	p.Arg121Trp	βB	Chapman et al. [2001] (family studies and absent in controls), Jackson et al. [2004]
ESDN-00234	MED	MED	*MATN3*	2	c.361C>T	p.Arg121Trp	βB	Chapman et al. [2001] (family studies and absent in controls), Jackson et al. [2004]
ESDN-00813	MED	MED	*MATN3*	2	c.361C>T	p.Arg121Trp	βB	Chapman et al. [2001] (family studies and absent in controls), Jackson et al. [2004]
ESDN-01071	MED	MED	*MATN3*	2	c.513_530 del	p.Asp171_Glu177delinsGlu	α4	In-frame deletion of functional residues
ESDN-00545^F^	Perthes	MED	*MATN3*	2	c.518C>A	p.Ala173Asp	α4	Fresquet et al. [2007] (absent in controls and biochemical analysis)
ESDN-00912	MED	MED	*MATN3*	2	c.584C>A	p.Thr195Lys	βD	Cotterill et al. [2005] (absent in controls)
ESDN-00774	MED	MED	*MATN3*	2	c.626G>C	p.Arg209Pro	α5	Co-segregation in four affected family members
ESDN-00065^Z^	MED	MED	*MATN3*	2	c.652T>A	p.Tyr218Asn	βE	Cotterill et al. [2005] (absent in controls) and co-segregation in this family
ESDN-01054	MED	MED	*MATN3*	2	c.656C>A	p.Ala219Asp	βE	Jackson et al. [2004] (family studies and absent in controls)
ESDN-00196^F^	MED	MED	*MATN3*	2	c.693G>C	p.Lys231Asn	α6	Fresquet et al. [2007] (absent in controls)
ESDN-00594^d^	MED	MED	*MATN3*	2	c.733G>A	p.Val245Met	βF	Bell et al. (unpublished manuscript submitted) (biochemical analysis)
ESDN-00638	MED	MED	*COL9A2*	3	c.186G>A	Skipping of exon 3	COL3	Holden et al. [1999] (family studies)
ESDN-01013	MED	MED	*COL9A2*	3	c.186G>A	Skipping of exon 3	COL3	Fielder et al. [2002] (family studies)
ESDN-00926	rMED	MED	*COL9A2*	3	c.186+2t>c	Skipping of exon 3	COL3	Muragaki et al. [1996] (family studies) and co-segregation in this family
ESDN-00997	MED	MED	*COL9A2*	3	c.186+2t>c	Skipping of exon 3	COL3	Muragaki et al. [1996] (family studies)
ESDN-01003	MED	MED	*COL9A2*	3	c.186+4a>c	Skipping of exon 3	COL3	Novel, but mutation co-segregates with affected mother and brother of the proband
ESDN-00986	MED	MED	*COL9A3*	3	c.148-2a>g	Skipping of exon 3	COL3	Novel, but c.148-2a>t shown to be pathogenic in Paassilta et al. [1999]

ESDN-00120	MED	rMED	*SLC26A2*	3	c.862C>T, c.862C>T	p.Arg279Trp, p.Arg279Trp	EC Loop 3	Ballhausen et al. [2003] (family studies)
ESDN-00121	MED	rMED	*SLC26A2*	3	c.862C>T, c.862C>T	p.Arg279Trp, p.Arg279Trp	EC Loop 3	Ballhausen et al. [2003] (family studies)
ESDN-00129	PPRD	rMED	*SLC26A2*	3	c.1984T>A, c.2171C>T	p.Cys653Ser, p.Ala715Val	TM 12/C-term	Rossi et al. [2001] Ballhausen et al. [2003] (family studies)
ESDN-00143	MED	rMED	*SLC26A2*	3	c.862C>T, c.862C>T	p.Arg279Trp, p.Arg279Trp	EC Loop 3	Ballhausen et al. [2003] (family studies)
ESDN-00167	PPRD	rMED	*SLC26A2*	1,3	−262t>c, c.794T>C	Splicing, p.Phe256Ser	5′ UTR/TM 5	Hastbacka et al. [1999] Novel (absent in controls)
ESDN-00189	Mild DTD	rMED	*SLC26A2*	3	c.862C>T, c.862C>T	p.Arg279Trp, p.Arg279Trp	EC Loop 3	Ballhausen et al. [2003] (family studies)
ESDN-00199	rMED	rMED	*SLC26A2*	3	c.862C>T, c.862C>T	p.Arg279Trp, p.Arg279Trp	EC Loop 3	Ballhausen et al. [2003] (family studies)
ESDN-00278	Mild SED	rMED	*SLC26A2*	3	c.862C>T, c.862C>T	p.Arg279Trp, p.Arg279Trp	EC Loop 3	Ballhausen et al. [2003] (family studies)
ESDN-00279	MED	rMED	*SLC26A2*	3	c.862C>T, c.862C>T	p.Arg279Trp, p.Arg279Trp	EC Loop 3	Ballhausen et al. [2003] (family studies)
ESDN-00292	MED	rMED	*SLC26A2*	3	c.862C>T, c.862C>T	p.Arg279Trp, p.Arg279Trp	EC Loop 3	Ballhausen et al. [2003] (family studies)
ESDN-00602	MED	rMED	*SLC26A2*	3	c.862C>T, c.862C>T	p.Arg279Trp, p.Arg279Trp	EC Loop 3	Ballhausen et al. [2003] (family studies)
ESDN-00634	rMED	rMED	*SLC26A2*	3	c.862C>T, c.862C>T	p.Arg279Trp, p.Arg279Trp	EC Loop 3	Ballhausen et al. [2003] (family studies)
ESDN-00700	(S)EMD	rMED/DTD	*SLC26A2*	3	c.862C>T, c.862C>T	p.Arg279Trp, p.Arg279Trp	EC Loop 3	Ballhausen et al. [2003] (family studies)
ESDN-00757	MED	rMED	*SLC26A2*	1,3	−262t>c, c.862C>T	Splicing, p.Arg279Trp	5′ UTR/Loop 3	Hastbacka et al. [1999] Ballhausen et al. [2003] (family studies)
ESDN-00970	AD MED	rMED	*SLC26A2*	3	c.862C>T, c.862C>T	p.Arg279Trp, p.Arg279Trp	EC Loop 3	Ballhausen et al. [2003] (family studies)
ESDN-01064	AD MED	rMED	*SLC26A2*	1,3	−262t>c, c.1984T>A	Splicing, p.Cys653Ser	5′ UTR/TM 12	Hastbacka et al. [1999] Rossi et al. [2001]

a Diagnosis as provided by the referring clinician.

b Consensus reached by the ESDN panel after review.

c Thought to be untypical MED for the following reasons; patient ESDN-00809 had unusual acetabula and flared iliac wings, there were also not typical metacarpal changes with proximal pointing, normal spine and “mouse-ear” ilia; patient ESDN-00049 had high vertebral bodies and rather slender tubular bones, unusually severe involvement of the proximal humerus, absence of epiphyseal abnormalities of the short tubular bones, and relatively normal carpal bones; patient ESDN-00323 had an unusual pelvic appearance and over modeled proximal tibia, proximal pointing of the metacarpals, quite generalized changes in the hand and carpal bones, and also significant changes in the spine. Nucleotide numbering according to cDNA sequence with nucleotide 1 counted as the first nucleotide of the translation initiation codon. GenBank accession numbers NM_000095.2 (*COMP*); NM_002381.4 (*MATN3*); NM_001852.3 (*COL9A2*); NM_001853.3 (*COL9A3*); NM_000112.3 (*SCL26A2*).

d Patient ESDN-00594 was shown to have a *MATN3* mutation (p.Val245Mat) in addition to two *COMP* mutations (p.Gly501Asp & p.Gln756Arg) and a polymorphism (c.2274+1g>c). The mutation in patient ESDN-00638 has previously been published (44).

^Z, F^These patients have previously been published in Zankl et al. [2007] and Fresquet et al. [2007], respectively.

Proof of pathogenicity is defined by one or more of the following criteria; (1) a previously published mutation with co-segregation in a family and/or absent in controls (as denoted in parenthesis), (2) a de novo mutation or co-segregation in this study, (3) alteration of an evolutionary conserved known functional residue in either the N-type motif or C-type motif of the type III repeat region of COMP, (4) biochemical evidence of pathogenetic affect.

PSACH, pseudoachondroplasia; MED, multiple epiphyseal dysplasia; SMD, spondylometaphyseal dysplasia; SEMD-JL, spondylo-epi-metaphyseal dysplasia with joint laxity; rMED, recessive form of MED; DTD, diastrophic dysplasia; PPRD, progressive pseudorheumatoid dysplasia; T3, type 3 repeat region of COMP; CTD, C-terminal domain of COMP; β-strand of matrilin-3 A-domain; α-helix of matrilin-3 A-domain; COL3, third collagenous domain of type IX collagen; EC, extracellular; TM, transmembrane regions of *SLC26A2*; n/d, diagnosis not discussed by ESDN.

*MATN3* mutations were identified in 13 MED patients and comprised predominantly of missense mutations (∼92%) and a novel in-frame deletion, all within exon 2 encoding the single A-domain of *MATN3* (Table 3). Nine mutations affected residues forming the internal β-sheet of the A-domain (i.e., βB, βD, βE, and βF), while four mutations affected residues in one of the six external α-helices (i.e., α4, α5, or α6) [Fresquet et al., 2007]. The recurrent mutations p.Thr120Met and p.Arg121Trp [Cotterill et al., 2005; Jackson et al., 2004] were each identified in more than one patient. We also identified an in-frame deletion/insertion (c.513_530del), which is predicted to result in a p.Asp171_Glu177delinsGlu in a single family with MED. MED patient ESDN-00594, who had previously tested heterozygous for p.Gly501Asp and p.Gln756Arg in *COMP*, was also shown to be heterozygous for p.Val245Met in *MATN3* (Tables 3 and 4).

**Table 4:** Two MED Patients with Multiple Mutations Identified in *COMP* and *MATN3*

ESDN 00359	Clinical status	*COMP* screening		
Proband	MED	c.1206T>C		
		c.1502G>A		
		c.1504G>T		
Father of proband	Unaffected	None detected		
Mother of proband	Affected	c.1206T>C		
		c.1502G>A		
		c.1504G>T		
Sister of proband	Unknown	None detected		

ESDN 00594	Clinical status	*COL9* screening	*MATN3* screening	*COMP* screening
Proband	MED	None detected	c.733G>A,p.Val245Met	c.1502G>A,p.Gly501Asp
				c.2267A>G,p.Gln756Arg
				c.2274+1G>C
Father of proband	MED or mild PSACH	n/a	c.733G>A,p.Val245Met	c.1502G>A,p.Gly501Asp
				c.2267A>G,p.Gln756Arg
				c.2274+1G>C

Patient ESDN-00359 was shown to be heterozygous for three *COMP* variants, which were all inherited from his affected father and therefore co-segregated as a single haplotype. Patient ESDN-00594 inherited three *COMP* variants and a single MATN3 variant from his affected father. Nucleotide numbering according to cDNA sequence with nucleotide 1 counted as the first nucleotide of the translation initiation codon. GenBank accession numbers NM_000095.2 (*COMP*); NM_002381.4 (*MATN3*).

Finally, we identified *COL9A2* mutations in five MED patients and a *COL9A3* mutation in a single MED patient (Table 3). The *COL9A2* mutations that we identified were all in the splice donor site of exon 3 and were therefore consistent with previous findings [Fiedler et al., 2002; Holden et al., 1999; Muragaki et al., 1996]. In our cohort of patients, mutations were identified at positions +3, +5, and +7 of the splice donor consensus sequence (i.e., ^+1^C C G g t g a g t^+9^) and are therefore consistent with those previously identified, with just one exception. MED patient ESDN-01003 was heterozygous for c.186+4a>c (i.e., +7 in consensus sequence), which has not been previously described. We did not directly assay whether this specific mutation would affect splicing, but the patient tested mutation negative for *COMP*, *MATN3*, *COL9A1*, and *COL9A3*, and the c.186+4a>c mutation co-segregated with the affected mother and brother of the proband.

Two patients had the relatively common c.186+2t>c change, which has previously been reported in several families of European origin [Muragaki et al., 1996]. We also identified a c.148-2a>g mutation in intron 2 of *COL9A3* in ESDN-00986. Although this specific sequence change has not been previously published, a c.148-2a>t mutation has been shown to be pathogenic and to result in the skipping of exon 3 of *COL9A3* due to the loss of the consensus “a” at the −2 position of a splice acceptor site [Paassilta et al., 1999].

In summary, we identified mutations in 27 patients with PSACH and 56 patients with AD-MED. The MED mutations that we identified in our patient cohort were found in the *COMP* (66%), *MATN3* (24%), *COL9A2* (8%), and *COL9A3* (2%) genes. We did not identify a *COL9A1* mutation in any patient sample analyzed. These data confirm recent studies showing that *COMP* mutations are the predominant cause of MED [Zankl et al., 2007], while type IX collagen gene mutations account for only about 10% of the currently known mutations in AD-MED.

### Mutation Analysis of SLC26A2 in AR-MED (rMED)

We screened 22 patients for mutations in *SLC26A2* that had a diagnosis consistent with AR-MED as determined by the ESDN expert panel. Sixteen (16/22, ∼73%) of these patients were either homozygous, or compound heterozygous, for *SLC26A2* mutations (Table 3). More specifically, of those 16 patients, 13 (13/15, ∼86%) were homozygous for the common p.Arg279Trp AR-MED mutation [Superti-Furga et al., 1999], while one patient was compound heterozygous (p.Arg279Trp and IVS1+2T>C). Three other patients were compound heterozygous, with the common “Finnish” mutation (IVS1+2T>C) [Hastbacka et al., 1999] and p.Cys653Ser both occurring twice; the remaining two mutations being p.Ala715Val and p.Phe256Ser. The latter mutation had not been observed prior to this study; its absence in well over 200 control samples and the proximity to two other known pathogenic mutations (p.Gly255Glu and p. Gly259Val) confirm its putative pathogenicity.

### Novel Mutations in the EGF-Like Repeats of COMP in PSACH and MED Patients

We failed to identify *COMP*, *MATN3*, *COL9A1*, *COL9A2*, *COL9A3*, or *SLC26A2* mutations in 30 patients that had originally been referred to ESDN with a working diagnosis of PSACH or MED and variants (Supp. Table S1). We therefore decided to extend the screening of some of these patients to include exons 1–7 of *COMP*, exons 3–6 of *MATN3*, exons 1–3 and 5–6 of *MATN1,* and exons 2 and 6+7 of *MATN4* (Supp. Table S1). We specifically chose these exons because they encode important structural and/or functional domains in glycoproteins that are structural components of the cartilage growth plate. For example, the EGF-like repeats of COMP and *MATN3* and the EGF-like and A-domains of matrilin-1 and -4 are important for protein integrity and interactions in cartilage [Wagener et al., 2005]. Moreover, mutations in the first EGF-like repeat of *MATN3* had been reported to cause recessive spondylo-epi-metaphyseal dysplasia (SEMD, *MATN3* related) [Borochowitz et al., 2004] and confer susceptibility to hand osteoarthritis [Stefansson et al., 2003].

**Figure 3:**
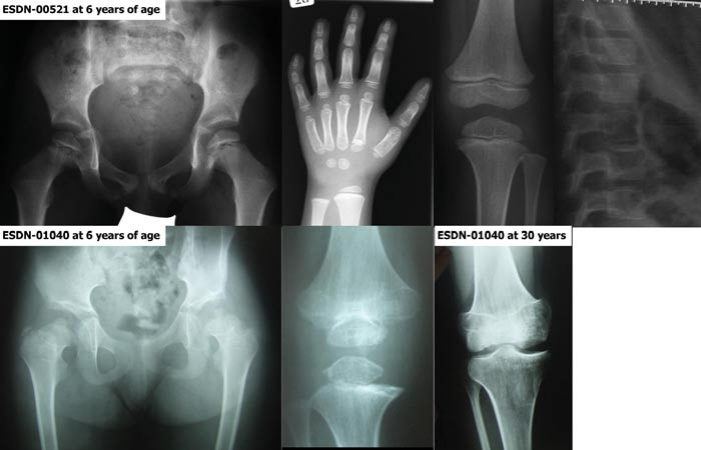
Radiographic findings in PSACH and MED patients with mutations identified in the EGF-like repeats of COMP. ESDN-00521: Radiographs taken at the age of 6 years. The proximal femoral epiphyses are small and flattened. The distal femoral and proximal tibial epiphyses are also small for age. There is no ossification yet of the proximal fibular epiphysis. The hand radiograph shows normal phalanges and metacarpals but delayed ossification of the carpal bones and epiphyses in the wrist. The spine is normal. ESDN-1040: The radiographs of pelvis and knee taken at the age of 6 years shows very small epiphyses in the hips and knees, which is reminiscent of pseudoachondroplasia. Note, also the two round translucent areas in the distal femoral metaphysis, which are often seen in patients with pseudoachondroplasia.

In one patient with MED and in the one remaining patient with “classical” PSACH, we identified novel mutations in exons 5 and 7 of *COMP*, respectively (Table 5; Fig. 3). MED patient ESDN-00521 was heterozygous for c.500G>A, which is predicted to result in a p.Gly167Glu substitution in the second EGF-like repeat of COMP. In PSACH patient ESDN-01040, we identified a heterozygous c.700C>T, which resulted in a p.Pro234Ser substitution in the fourth EGF-like repeat of COMP; both of these unclassified variants had not previously been reported and were not present in the dbSNP database version 130 (May 2009). In contrast, no mutations were identified in the additional exons of *MATN1*, *MATN3*, and *MATN4* that we screened, which is consistent with our previous studies [Jackson et al., 2004].

**Table 5:** Novel *COMP* and *COL2A1* Mutations Identified in Four Patients with Clinically and Radiographically Confirmed PSACH or MED

Patient	Phenotype	Gene	Exon	Nucleotide change	Protein change	Domain	Proof of pathogenicity
ESDN-00521	MED	*COMP*	5	c.500G>A	p.Gly167Glu	EGF-like 2	Evolutionally conserved functional residues in type II repeats of COMP and not present in 20 other PSACH-MED patients
ESDN-01040	PSACH	*COMP*	7	c.700C>T	p.Pro234Ser	EGF-like 4	

ESDN-00050	MED	*COL2A1*	50	c.3535G>A	p.Gly1179Arg	Triple helical region	Highly conserved glycine residues vital for correct triple helical formation. Mutations in neighboring glycine residues, p.Gly1173Arg and p.Gly1176Ser, shown to be pathogenic
ESDN-00283	MED	*COL2A1*	50	c.3527G>T	p.Gly1176Val	Triple helical region	

COMP EGF-like mutations were identified in MED patient ESDN-00521 and PSACH patient ESDN-01040, both of which are novel variants that are not present in the dbSNP database version 130 (May 2009). *COL2A1* mutations identified in two MED patients (ESDN-00050 and -00283) that affected conserved glycine residues in the triple helical region of type II collagen. Nucleotide numbering according to cDNA sequence with nucleotide 1 counted as the first nucleotide of the translation initiation codon. GenBank accession numbers NM_000095.2 (*COMP*); NM_001844.4 (*COL2A1*).

### Novel Mutations in Exon 50 of COL2A1

Finally, we extended our screening to include exon 50 of *COL2A1* since a recurrent mutation (p.Gly1170Ser) in this exon has been shown to cause Legg-Calve-Perthes (LCP) disease in four families [Miyamoto et al., 2007; Su et al., 2008], and there is clear clinical overlap between LCP and MED [Herring and Hotchkiss, 1987; Ikegawa et al., 1991]. Patient ESDN-00050 (mild MED) was heterozygous for c.3535G>A, which is predicted to result in a p.Gly1179Arg substitution and patient ESDN-00283 (MED) was heterozygous for c.3527G>T, which is predicted to result in a p.Gly1176Val substitution (Table 5).

## Discussion

In this study, we undertook a comprehensive clinical and molecular approach to define the genetic basis of PSACH and MED in a cohort of 130 patients referred to ESDN. All of these patients had been referred to ESDN between 2003 and 2010 with various working diagnoses of PSACH (27); PSACH-MED (3); MED (66) (including variants described as Fairbank (2), polyepiphyseal dysplasia (1), MED with sacroiliitis (1), MED with neuropathy (1) and Perthes (2)); rMED (9); spondyloepiphyseal dysplasia (SED)/MED (1); SED (4); SEMD (2) (including a variant described as SEMD-JL [1]); SMD (1); PPRD (2); mild DTD (3), or without any formal diagnosis (4).

For the vast majority of patients referred to ESDN with a provisional diagnosis of PSACH, the panel agreed with the diagnosis and a *COMP* mutation was subsequently found (Table 1). This observation implies that PSACH is relatively straightforward to diagnose given suitable radiographs and clinical summary, however, it should be noted that at least 21 of the 27 PSACH referrals came from geneticists within clinical genetics departments, including eight referrals from members of the ESDN panel, suggesting that the cases came from clinicians with experience in skeletal dysplasias. The three exceptions were ESDN-00074, ESDN-00618, and ESDN-00695, but atypical clinical and radiographic features excluded PSACH and suggested an alternative diagnosis prior to sequencing the COMP gene (Table 2; Fig. 2).

In the other 56 patients in whom we identified a *COMP*, *MATN3* or type IX collagen gene mutation, the majority had been referred to ESDN with a diagnosis of MED (Table 3), which would indicate that the “classical” forms of MED (i.e., those patients in whom we identified a mutation) are also relatively easy to diagnose. This was particularly the case for those patients in whom we identified *MATN3*, *COL9A2*, or *COL9A3* mutations [i.e., 18/19 (94%) patients with these mutations had a correct diagnosis upon referral to ESDN; see Table 3]. Once again these referrals came almost exclusively from geneticists within clinical genetics and/or pediatrics departments and included nine referrals from members of the ESDN panel.

Finally, in those patients in which we identified a *DTDST* mutation, 69% (11/16 patients) had originally been referred with a diagnosis of MED or rMED; with PPRD (2) and mild DTD/SED/SEMD (3) being proposed as a diagnosis in five cases. This observation would therefore suggest that rMED is slightly harder to diagnose than classical AD-MED. This may be explained partly by the fact that unlike AD-MED where family history is often positive, most cases of rMED lack a family history and therefore physicians may be less inclined to think of a possible genetic cause of the disorder. Likewise, all the referrals came from geneticists within clinical genetics departments and also included three referrals from members of the ESDN panel.

In spite of the diagnostic difficulties, we can confirm that autosomal recessive MED (rMED) accounts for approximately one-fourth of total MED cases, as has been suggested by earlier studies (15, 16). This relatively high incidence is driven by the frequency of the p.R279W mutation, which is by far the most common *DTDST* mutation in the European population [Ballhausen et al., 2003; Barbosa et al., 2010; Rossi and Superti-Furga, 2001]. Interestingly, it has been reported that *DTDST* mutations are not a common cause of MED in some Asian populations [Itoh et al., 2006]. On the whole, this study suggests that ESDN receives a significant number of referrals from geneticists and/or pediatricians with an interest in, and knowledge of, skeletal dysplasias, which is reflected in the relatively high level of correct diagnosis on referral.

**Figure 4:**
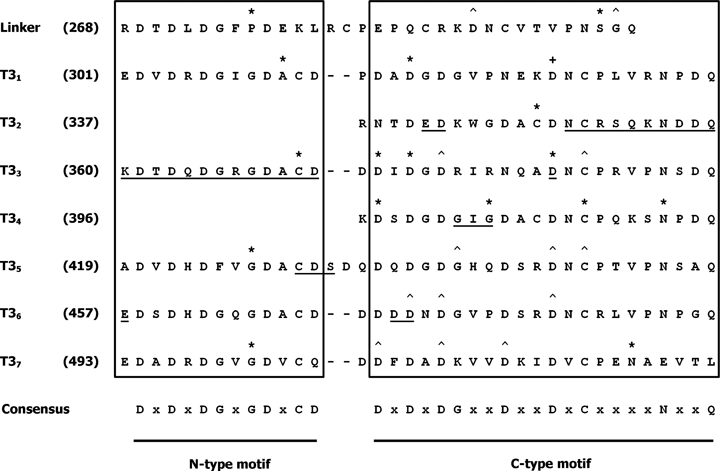
Localization of *COMP* mutations identified in PSACH and MED patients in this study. An amino acid sequence alignment of the type III repeats region of COMP, with the linker region and each of the seven T3 repeats (T3_1-7_) shown with their corresponding residue numbers. Residues comprising the N-type and C-type motif are boxed and the consensus sequence of each motif is indicated below. Also shown on the alignment are the missense mutations that cause either MED (*), PSACH (^), or both (+) phenotypes. In-frame deletions are underlined.

The range of *COMP* mutations that we identified in the PSACH patients was similar to those previously published [Kennedy et al., 2005a] and included missense mutations that resulted in the substitution of conserved glycine, aspartic acid, asparagine, and cysteine residues, which are important for the folding, structural integrity, and calcium binding of the type III repeats [Tan et al., 2009]. We also identified the common p.Asp473del mutation in six PSACH patients and more complex deletions in three other patients, thus in-frame deletions were identified in approximately 33% of PSACH patients (9/27), which was slightly less than the 43% that we have previously reported [Kennedy et al., 2005a]. The range of *COMP* mutations that we identified in the MED patients was more diverse than those found in PSACH and in addition to the substitution of conserved glycine, aspartic acid, asparagine, and cysteine residues, we also identified missense mutations that resulted in the substitution of nonconserved proline (p.Pro276Arg) and serine (p.Ser298Leu) residues and a conserved alanine (p.Ala311Asp) residue all within the linker or the T3_1_ repeat of the Type III region (Fig. 4). We also identified a broad range of in-frame deletion, duplication, and deletion/insertion mutations including the previously reported p.Asp473dup [Delot et al., 1999]. It is an interesting observation that p.Asp473del consistently causes PSACH, while p.Asp473dup always causes MED. Presumably, the insertion of a aspartic acid reside into the C-type motif of T3_6_ is less deleterious to protein folding and structure than its deletion.

Interestingly, we identified the recurrent p.Asn523Lys (c.1569C>G) mutation in three MED patients from our panel (ESDN-00123, 00382, and 00751), all of whom were from the Netherlands. Furthermore, this same mutation was previously identified in a large South African kindred of Dutch descent [Ballo et al., 1997], suggesting that this is an ancestral mutation. We also identified the recurrent p.Asp385Asn (c.1153G>A) mutation in two British and one Dutch family with MED (ESDN-00049, 00509, and 00597), in addition to p.Asp585Asn and p.Asp385del mutations, which points to a key role for Asp385 in the structure of COMP.

Finally, when just considering the 34 different *COMP* missense mutations that we identified in the type III region, sequence alignments reveal that 85% (29/34) of them affect residues in the C-type motif of the linker and T3_1-7_ repeats (Fig. 4). This suggests that conserved residues in this motif are more important for coordinating calcium binding and/or the folding of COMP; the five mutations that we identified in the N-type motif all cause MED.

Mutations in exons 14–18 of *COMP*, which encodes the C-terminal domain were identified in approximately 13% (8/64) of PSACH and MED patients and were once again clustered at specific residues as previously noted [Kennedy et al., 2005b]. These observations reinforce the hypothesis that Thr529, Thr585, Arg718, and Gly719 are important for the structure and/or function of the C-terminal domain of COMP. Indeed, we have now identified every possible amino acid substitution of Thr585 [i.e., pThr585Met, p.Thr585Lys (unpublished data), and p.Thr585Arg], which all result from either a transversion or transition at nucleotide c.1754, suggesting that this nucleotide is particularly susceptible to mutation, but also confirming that methionine at residue 585 is vital for the correct folding and/or functioning of COMP.

MED mutations in *MATN3* were again found in exon 2, which encodes the single A-domain of *MATN3* and affected residues in either the β-stands (70%) or α-helices (30%). The identification of an in-frame deletion/insertion (c.513_530del), which is predicted to result in a p.Asp171_Glu177delinsGlu in the α4 helix, is the first mutation of this kind to be identified in *MATN3* and thus extends the type of mutations in *MATN3* that can cause MED. The clustering of mutations in exon 2 demonstrates the importance of the A-domain and most studies have demonstrated that these mutations disrupt the folding of this domain [Cotterill et al., 2005], which elicits and unfolded protein response [Nundlall et al., 2010].

Interestingly, in all six patients in whom we identified a *COL9* mutation, the ESDN panel had predicted or suggested a type IX collagen defect prior to mutation screening. The ability of the panel to accurately predict a *COL9* mutation was a result of the previously documented differences in the clinical and radiographic presentation of MED caused by a type IX collagen mutation compared to *COMP* and *MATN3* mutations [Lachman et al., 2005; Unger et al., 2008, 2001]. *COL9*-MED is generally the mildest form of MED and is characterized by joint pain and stiffness presenting in the first decade of life, while radiographic abnormalities are primarily restricted to the knees with relative sparing of the hips. Interestingly, three of the six patients (ESDN-0638, 0926, and 0997) were from the Netherlands and two of these shared the same c.186+2C>T mutation in *COL9A2*, which was originally identified in a large Dutch kindred in 1986 [Muragaki et al., 1996; van Mourik et al., 1998] and more recently in a second large family of Dutch origin [Jackson et al., 2010; Versteylen et al., 1988]. These data might suggest that the c.186+2t>c mutation in *COL9A2* is another ancestral mutation in the Dutch population; however, haplotype analysis should be performed to test this hypothesis further. In contrast, three British families with *COL9A2*-MED all had a different mutation; c.186+4a>c (ESDN-01003 in this study), c.186+5g>c [Holden et al., 1999], and c.186+6t>g [Barrie et al., 1958; Briggs et al., 1994; Spayde et al., 2000]. Finally, although it has been proposed that *COL9*-MED mutations are more prevalent in Japan [Itoh et al., 2006], it is interesting to note that of the 14 *COL9A2* mutations published to date, 12 have actually been identified in families from Northern Europe (UK [3], Netherlands [4], Germany [2], Sweden [1], and unspecified [1]). Our study has now demonstrated that mutations in *COL9A2* and *COL9A3* account for approximately 10% of mutations in molecularly confirmed MED, which is slightly less than the 16% reported by Itoh and colleagues [Itoh et al., 2006]. In contrast, *COMP* mutations accounted for 66% of mutations (37% in [Itoh et al., 2006]) and *MATN3* for 24% of mutations (47% in [Itoh et al., 2006]). These differences in the relative proportions of *COMP*, *MATN3*, and *COL9* mutations may be due to ascertainment bias or ethnic differences.

The panel's success in predicting a *COL9* mutation was not repeated with MED resulting from *MATN3* mutations and in most cases the panel could not decide between *COMP* or *MATN3* as the causative gene prior to screening (although the *COL9* genes were never considered as candidates). These observations would suggest that there are phenotypic features (both clinical and radiographic) shared between *COMP*-MED and *MATN3*-MED, which may result from common disease mechanisms. Indeed, recent studies of knock-in mouse models of mild PSACH and MED caused by *Comp* and *Matn3* mutations, respectively, suggest that specific characteristics of growth plate pathophysiology, such as reduced chondrocyte proliferation and increased and/or spatially dysregulated apoptosis are common disease mechanisms [Leighton et al., 2007; Pirog-Garcia et al., 2007].

Interestingly, in two unrelated patients with AD-MED, we identified more than one potential mutation that co-segregated with the phenotype following family studies (Table 4). MED patient ESDN-00359 had three changes identified in *COMP*; p.Gly404Gly (a nonpathogenic neutral polymorphism), p.Gly501Asp, and p.Asp502Tyr, which are both conserved amino acid residues in the N-type motif of the T3_7_ repeat and help to co-ordinate Ca^2+^ binding. All three changes co-segregate as a single haplotype since they were also present in the suspected affected mother, but not the unaffected father, of the proband. In this case, it would seem most likely that a single mutational event affecting contiguous nucleotides in the codons of both Gly501 (GGC) and Asp 502 (GAC) would account for these two mutations (p.Gly501Asp and p.Asp502Tyr). Both mutations might conceivably cause MED on their own by affecting highly conserved amino acids important for protein folding and calcium binding, respectively.

ESDN-00594 had four changes; three in *COMP* and a single change in *MATN3*, none of which have previously been reported (Table 4). The COMP changes included p.Gly501Asp (also identified in ESDN-00359 and ESDN-00422); p.Gln756Arg, which is at the end of the C-terminal domain of COMP (and interestingly arginine is present at that same position in bovine and rat COMP); and finally c.2274+1G>C which is in the 3′ untranslated region and might affect mRNA stability leading to reduced protein levels, but since COMP-null mice are normal, this change is unlikely to have a phenotypic effect. The *MATN3* mutation is at a conserved residue (p.Val245) in the αF strand of the *MATN3* A-domain and functional studies show p.Val245Met affects to some extent the trafficking and secretion of *MATN3* A-domain [unpublished data]. Importantly, the p.Gly501Asp mutation in ESDN-00422 was recently confirmed as de novo in this family [Stephen Robertson, personal communication], confirming that it is the cause of MED in ESDN-00594. Nevertheless, the intracellular retention of a significant proportion of p.Val245Met suggests the intriguing possibility that it might be a genetic modifier of phenotypic severity.

By extending our standard screening protocol, we identified mutations in *COMP* and *COL2A1* in the remaining PSACH patient and in three MED patients. In PSACH patient ESDN-01040, we identified a heterozygous p.Pro234Ser substitution in the fourth EGF-like repeat of COMP and MED patient ESDN-00521 was heterozygous for p.Gly167Glu in the second EGF-like repeat of COMP. Both of these residues are conserved in murine COMP and the substitution of glycine and proline residues in the EGF-like repeats of fibrilin-1 has been shown to cause Marfan Syndrome [Arbustini et al., 2005; Collod-Beroud et al., 1999]. More recently, we have identified a third *COMP* EGF-like mutation in a patient with PSACH, p.Gly258Arg, which is in the fourth repeat and again conserved across species (unpublished data), but the precise affect of these *COMP* mutations remains undetermined and will require extensive studies in vitro.

Both of the *COL2A1* mutations that we identified (p.Gly1176Val and p.Gly1179Arg) were in suspected MED patients (ESDN-00283 and ESDN-00050) in whom there was limited clinical information and radiographic images in which to make an unambiguous diagnosis (Table 5). However, in both cases while there were some features consistent with MED, it was also noted that there were features not normally associated with MED such as short trunk and severely fragmented hip epiphyses with adjacent metaphyseal anomalies. This observation would suggest that there is some clinical and radiographic overlap between MED and mild SED congenital (SEDc), which is borne out by the fact that similar mutations, p.Gly1173Arg [Sobetzko et al., 2000] and p.Gly1176Ser [Williams et al., 1995], have previously been shown to cause SEDc type. Furthermore, the identification of a recurrent p.Gly1170Ser mutation in patients with Legg-Calve-Perthes disease (LCPD) and/or primary avascular necrosis of femoral head (ANFH) [Miyamoto et al., 2007; Su et al., 2008] also suggests that there are similar disease mechanisms that might cause phenotypes within a LCPD/ANFH-MED-SEDc disease spectrum.

Finally, in those patients in whom we could not identify a mutation in the core exons of our screening protocol, the predominant diagnosis on referral had been MED or rMED (Supp. Table S1: 22/30 [80%]), with the remainder being PSACH (1), DTD (2), SED (3), or unknown (2). This observation would suggest that the mutation negative cases of MED are due to either a mutation in an as yet unknown gene(s), or the diagnosis of MED was incorrect in these patients. Interestingly, on re-review of these cases it was clear that the ESDN panel had not agreed upon a consensus diagnosis for most of these cases. Indeed, there were only two mutation negative patients in which a diagnosis of suspected mild MED was agreed upon by the panel prior to screening (Supp. Table S1; ESDN-00039 and ESDN-00160). In the majority of cases in which we did not identify a mutation in this study (Supp. Table S1: 24/26 [96%]), an alternative diagnosis was suggested prior to mutation screening and for many cases this was either Meyer's disease/hip dysplasia (Beukes)/bilateral LCPD (8/26), a type II collagenopathy (5/26), or SEMD (2). This would suggest that there are forms of familial hip dysplasia, variably described in the literature as Meyer's disease (dysplasia epiphysealis capitis femoris), familial hip dysplasia (Beukes), and bilateral LCPD, that are genetically distinct from the classical forms of MED and do not result from mutations in COMP, *MATN3*, or type IX collagen. The genetic cause of these diseases remains underdetermined, but the careful use exome sequencing may help identify potential candidate genes. Finally, it is interesting to note that like the mutation positive cases, the majority of mutation negative patients had also been referred by geneticists within clinical genetics departments including six from members of the ESDN panel. This would indicate that in these patients there are clear difficulties in making a correct diagnosis rather than a lack of relevant expertise.

In summary, we have shown that in the context of PSACH and the MED disease spectrum, the classical form of PSACH is relatively straightforward to diagnose provided there is sufficient clinical and radiographic information. In cases of PSACH, a *COMP* mutation should be identified, however, we have additional evidence to confirm that a PSACH-like phenotype is distinct from classical PSACH and does not result from a *COMP* mutation [Spranger et al., 2005]. In contrast, the radiographic signs of MED are more subtle and variable, and while the ESDN panel was relatively successful in predicting genotype from the phenotype, MED remains more difficult to diagnose correctly. Our study confirms that accurate review by an expert panel may help in prioritizing the genes to be sequenced and thus reduce both time and cost. Those cases that remain “mutation negative” should be carefully re-reviewed and alternative diagnoses possibly considered. Finally, our comprehensive study also throws doubt on previous studies that have suggested that mutations in the known genes are not the major cause of MED [Jakkula et al., 2005], and we conclude that mutations in *COMP*, *MATN3*, and type IX collagen genes account for the vast majority of classical AD-MED.
