# Supplementary material for: Pseudoachondroplasia and Multiple Epiphyseal Dysplasia: A 7-Year Comprehensive Analysis of the Known Disease Genes Identify Novel and Recurrent Mutations and Provides an Accurate Assessment of Their Relative Contribution
Source: Hum Mutat. 2011 Sep 15;33(1):144–57. doi: 10.1002/humu.21611 (PMC3272220; doi:10.1002/humu.21611)
Supplement: Supplementary file 1 [file humu0033-0144-SD1.pdf]

Supp. Table S1. 30 patients referred to ESDN with a working diagnosis of MED in which mutations were not identified in the core exons screened

Patient	Diagnosis on referral [1]	Genes screened	Reasons why not 'classical' MED [2]	Alternative diagnosis suggested prior to mutation screening [3]
**ESDN-00018**	MED	1 -10	1) Fragmented capital femoral epiphyses<br>2) Slightly flattened vertebral bodies and knee epiphyses	1) Meyer's disease<br>2) Bilateral Legg-Calvé-Perthes<br>3) Beukes hip dysplasia
**ESDN-00022**	MED	1-10	1) Severe osteoporosis with sequelae of fractures on the forearm	1) Bilateral Legg-Calvé-Perthes<br>2) Hip dysplasia
**ESDN-00039**	MED Fairbanks type	1-10	1) Very mild radiographic features with normal hips	None suggested
**ESDN-00045**	BPD/MED	1-11	1) Metaphyseal involvement<br>2) Short femoral necks	1) Meyer's disease<br>2) Beukes hip dysplasia
**ESDN-00047**	MED	1-11	1) Double-layered patella	1) Meyer disease
**ESDN-00050**	MED	1-11	None described	None suggested
**ESDN-00088**	MED	1-11	1) Hips mainly involved with metaphyseal abnormalities of proximal femoral growth plates<br>2) Wide 5th metacarpal	1) Meyer's disease<br>2) Bilateral Legg-Calvé-Perthes
**ESDN-00095**	rMED	6	1) Abnormal pelvis with small iliac wings, horizontal acetabular roofs and short femoral necks<br>2) Slightly short femoral necks, small ilea, short metacarpals, advanced bone age, relatively normal proximal femoral epiphyses.<br>3) No double-layered patellae, small flattened capital femoral epiphyses. Short phalanges and metacarpals, advanced carpals, short broad femoral necks and mild changes in the spine.	1) Type II collagenopathy
**ESDN-00101**	DTD?	6	1) Some residua of coronal clefting. In addition small iliac wings, horizontal acetabular roofs, short femoral necks, small proximal femoral and proximal tibial epiphyses, *genu valga* (and not *vara*?) and club feet	1) Mucopolysaccharidosis type IVB (Morquio's syndrome)
**ESDN-00110**	MED	1-10	1) Minor asymmetry of the capital femoral epiphyses<br>2) Vertebral bodies are slightly flattened and pear-shaped	1) Bilateral Legg-Calvé-Perthes<br>2) Mild SED
**ESDN-00118**	rMED	6	1) Hand X-ray not typical due to retarded bone age<br>2) The appearance of the inter-pediculate distance is not typical	1) Mild spondyloepiphyseal dysplasia Omani type

Patient	Diagnosis on referral [1]	Genes screened	Reasons why not 'classical' MED [2]	Alternative diagnosis suggested prior to mutation screening [3]
**ESDN-00146**	Diastrophic dysplasia	6	None described	1) Stüve-Wiedemann (Schwartz-Jampel syndrome type 2)
**ESDN-00156**	MED	1-11	1) Marked changes in the spine and metaphyseal and epiphyseal changes.	1) SEMD
**ESDN-00160**	MED	1-10	1) Little radiographic documentation	None suggested
**ESDN-00283**	MED	1-5, 7-10	1) Little radiographic documentation<br>2) Short trunk<br>3) Abnormal metaphyses	None suggested
**ESDN-00521**	MED	1-10	1) Widening and irregularity of the upper femoral metaphyses<br>2) Epiphyses not as flattened as they are in typical cases	1) Spondyloepiphyseal dysplasia Omani type
**ESDN-00603**	AD MED	1-5, 7-11	1) Mild scoliosis and normal vertebrae on lateral spine<br>2) Only very mild radiographic changes	1) Familial predisposition to premature arthrosis
**ESDN-00741**	MED	1-5, 7-11	1) Serrated iliac crests<br>2) Bony erosion/reabsorption (carpals; distal metacarpals; prox phalanges subluxated!; prox tibias; elbows)<br>3) Distally scooped out metacarpal epiphyses, crowded and irregular carpal bones and crenated iliac crests	1) Osteolysis variant or a rheumatoid variant<br>2) Erosive arthropathy
**ESDN-00756**	Unknown	1-5, 7-10	1) Short-trunk<br>2) Normal hands spine, flat capital femoral epiphyses, distinct shortness of stature	1) Mild type II collagenopathy
**ESDN-00843**	rMED	1-6	1) Absence of double layered patella<br>2) Unusual hands	1) Type II collagenopathy
**ESDN-00845**	rMED	6	1) N- and O- glycosylation defect	1) Roifman Syndrome
**ESDN-00861**	SEDc	1-5, 7-11	1) Teeth abnormalities<br>2) Dysmorphic face and hearing loss	1) Mild CODAS<br>2) Saul-Wilson
**ESDN-00876**	AD MED	1-5, 7-11	None described	1) Type II collagenopathy
**ESDN-00879**	SED	1-5	1) Myopia<br>2) No vertebral changes	1) Type II collagenopathy<br>2) Familial precocious osteoarthritis
**ESDN-00885**	rMED	1-5	1) Fragmented femoral epiphyses	1) Dysplasia epiphysealis femoris
**ESDN-00989**	AD MED	1-5	1) Severely affected capital femoral epiphyses	1) Bilateral Legg-Calvé-Perthes<br>2) Aarskorg Syndrome

Patient	Diagnosis on referral [1]	Genes screened	Reasons why not 'classical' MED [2]	Alternative diagnosis suggested prior to mutation screening [3]
**ESDN-01039**	Unknown	1-5	1) Mild delay in bone maturation but not MED	1) Thyroid abnormality
**ESDN-01040**	PSACH	1-5, 7-11	None described	None suggested
**ESDN-01068**	AD MED	1-5	1) Little radiographic documentation	1) Bilateral Legg-Calvé-Perthes
**ESDN-01102**	SED tarda (X-linked)	1-5, 7-11	1) Delicate middle/distal phalanges and multiple ivory epiphyses	1) 'Syndromic' MED<br>2) Unclassified (S)MED<br>3) Conradi-Hunermann

A summary of the radiographic and/or clinical feature of 30 patients referred to ESDN with a working diagnosis of MED (or another disease) in which a mutation was not identified in the core exons of the screening protocol. [1] Diagnosis as provided by the referring clinician; [2] The key clinical and/or radiographic features why the ESDN panel felt this was not a case of classical MED; [3] Alternative diagnoses suggested after review of the case and prior to commencing mutation screening. Additional genes screened = [1] *COMP* exons 8-19; [2] *COL9A1* exon 8; [3] *COL9A2* exon 3; [4] *COL9A3* exon 3; [5] *MATN3* exons 2; [6] *DTDST* exons 1-3; [7] *COMP* exons 1-7; [8] *MATN3* exons 3-6; [9] *MATN1* exons 1-3 and 5-6; [10] *MATN4* exons 2 and 6-7; [11] *COL2A1* exon 50. Key: PSACH = pseudoachondroplasia; BPD = bilateral Perthes disease; MED = multiple epiphyseal dysplasia; rMED = recessive form of MED; DTD = diastrophic dysplasia; SED = spondyloepiphyseal dysplasia; SEMD = spondylo-epi-metaphyseal dysplasia; CODAS = Cerebral, Ocular, Dental, Auricular, Skeletal anomalies syndrome (MIM# 600373).

Supp. Table S2. Primers used for this study

Gene	Exon	Forward primer	Reverse primer
COL2A1	50	5’ ctccccagccttccctgt 3’	5’ ggatgccatcactgttag 3’
MATN3	3	5’ aaaggagcccagagagcaat 3’	5’ cagtccaaaacctggagcat 3’
MATN3	4	5’ cgtggcccacaattatttc 3’	5’ cacaccaacttcccagtcaa 3’
MATN3	5	5’ tccagctgcaaataaggtttc 3’	5’ ttgcaagttggtttcatgtg 3’
MATN3	6	5’ ggaagagaaaacaggcttgc 3’	5’ tgacagggagaaagaatcacaa 3’
MATN1	1	5’ ataagacctggaccccactg 3’	5’ gtgtgctccctgccatatc 3’
MATN1	2	5’ gcctttggcaagttgctt 3’	5’ cctgtttccccagctatgaa 3’
MATN1	3	5’ gaaacgggtctcctcttcg 3’	5’ ttctatctctctcccgtctcc 3’
MATN1	5	5’ gggagcaggatggttgacta 3’	5’ ggcctcaattttgtcattgg 3’
MATN1	6	5’ gtccttgttccaggcattgt 3’	5’ cacacccagttccaggatg 3’
MATN4	2	5’ tgggtaataccgcctcagaa 3’	5’ aggttgctccttgttgatgg 3’
MATN4	6 + 7	5’ aaatgtttgcgaatgaacga 3’	5’ tctgattcattcggtctgga 3’
COMP	1	5’ cagcgccatctgtttacctt 3’	5’ tctcacgggtcctacagtcc 3’
COMP	2	5’ caacggtgggagttggtg 3’	5’ tccaccttctcgggtacttc 3’
COMP	3	5’ gggtagagagaggggagacc 3’	5’ ctcttttcctccccagcttt 3’
COMP	4	5’ ggaaggggaagcctccag 3’	5’ gaacactcccagtggaggaa 3’
COMP	5	5’ gtcatcaaggcaaagtcgtg 3’	5’ ggaggctggaagaggagttt 3’
COMP	6 + 7	5’ gaggaagtgtggaggaggtg 3’	5’ gtaagtgggtgccctggagt 3’
